# The signaling involved in autophagy machinery in keratinocytes and therapeutic approaches for skin diseases

**DOI:** 10.18632/oncotarget.9330

**Published:** 2016-05-12

**Authors:** Li Li, Xu Chen, Heng Gu

**Affiliations:** ^1^ Institute of Dermatology, Chinese Academy of Medical Science & Peking Union Medical College, Jiangsu Key Laboratory of Molecular Biology for Skin Diseases and STIs, Nanjing, China

**Keywords:** autophagy, keratinocyte, skin, skin disease, autophagy-related gene

## Abstract

Autophagy is responsible for the lysosomal degradation of proteins, organelles, microorganisms and exogenous particles. Epidermis primarily consists of keratinocytes which functions as an extremely important barrier. Investigation on autophagy in keratinocytes has been continuously renewing, but is not so systematic due to the complexity of the autophagy machinery. Here we reviewed recent studies on the autophagy in keratinocyte with a focus on interplay between autophagy machinery and keratinocytes biology, and novel autophagy regulators identified in keratinocytes. In this review, we discussed the roles of autophagy in apoptosis, differentiation, immune response, survival and melanin metabolism, trying to reveal the possible involvement of autophagy in skin aging, skin disorders and skin color formation. Since autophagy routinely plays a double-edged sword role in various conditions, its functions in skin homeostasis and potential application as a therapeutic target for skin diseases remains to be clarified. Furthermore, more investigations are needed on optimizing designed strategies to inhibit or enhance autophagy for clinical efficacy.

## OVERVIEW OF AUTOPHAGY AND AUTOPHAGY MACHINERY

Autophagy is a conserved catabolic process in which cellular constituents including proteins, organelles and invaded microorganisms are captured and targeted for lysosomal degradation. The process is initiated by generation of the double-membrane autophagosomes which then fuse with lysosomes to form autolysosomes where all contents are enzymatically digested. Autophagy is involved in a large range of cell physiological processes, including immune response, tumorigenesis, differentiation, apoptosis, anti-microbial defense etc. [1-3]. Disordered autophagy has been reported to be associated with a wide range of human diseases such as neurodegenerative diseases, cardiomyopathies, infectious diseases and cancers, and autophagy modulation has been already considered as a potential target for therapy of these diseases [4, 5]. Activation of autophagy is characterized by specific intracellular biochemical changes. Nearly forty autophagy-related (ATG) proteins have already been identified to participate in different steps of autophagy process, including the recognition of target cargo, and formation, migration, fusion and maturation of autophagosomes [6-8].

Mammalian target of rapamycin (mTOR) is the key up-stream regulator of autophagy, and reduction of mTOR activity is usually accompanied by enhanced phosphorylation of AMP Kinase (AMPK) under metabolic stresses. AMPK, a key energy sensor, can promote autophagy through interacting with tuberous sclerosis complex 2 (TSC2) heterodimer, mTOR complex 1 (mTORC1) subunit raptor or UNC-51-like kinase 1 (ULK1) (homolog of yeast ATG1) [9-11]. Two upstream kinases, Liver kinase B1 (LKB1) and calcium/calmodulin-dependent protein kinase (CaMK) are responsible for activating AMPK [12, 13]. Lower energy state could trigger AMPK phosphorylation and subsequent inhibited mTORC1 accompanied with dephosphorylation of p70 S6 kinase (p70S6K) [14, 15].

Two ubiquitin-like conjugation systems of ATG5–ATG12 protein complex and microtubule-associated protein light chain 3 (LC3) are essential for the process of autophagosome formation. ATG5 conjugates with ATG12, an ubiquitin-like molecule, by a series of ubiquitination-like reactions involving ATG7 and ATG10 [16]. ATG5-ATG12 then conjugates with ATG16L forming ATG5–ATG12–ATG16L1 complex which is essential for the initiation of phagophore elongation [17]. LC3 is a specific component of autophagosome membrane. Precursor of LC3 protein synthesized in the cytoplasm is converted to LC3-I form through cleaving carboxy terminus by cysteine protease ATG4, and then its glycine residue is exposed for E1-like enzyme ATG7. Finally, LC3-I is converted to LC3-II through conjugating with phosphatidylethanolamine (PE) under catalysis of ATG7 and ATG3 [18]. Notably, ATG7 is a key regulator for autophagosome formation and one of the most important ATG members, based on which many animal models are designed and established to investigate the role of autophagy in regulating cell physiological processes. The Rac1-Armus-Rab7 axis was recently reported to participate in regulating recruitment of LC3 to autophagosomes in keratinocytes [19]. TBC/RabGAP Armus, especially inactivates Rab7, is an effector of Rac1, a small GTPase involving in a wide range of cellular function regulation including cell adhesion, migration, survival and mitosis and cytokinesis, etc. [20-22]. Inactivated Armus delays autophagy flux by blocking the initiation of phagophore, while Rab7 is transiently activated. Activated Rac1 competes with LC3 for Armus to prevent its recruitment to autophagosomes. Intracellular components targeted for autophagy-related degradation are sequestered and bound to LC3 by adaptor proteins like p62/SQSTM1, neighbor of BRCA1 gene 1 (NBR1) protein and autophagy-linked FYVE protein (ALFY) [23-25]. Besides, Beclin1, a component of Class III phosphatidylinositol 3-kinase (PtdIns3K) complex, is another key modulator that must be mentioned. Beclin1 and PtdIns3K are collaboratively involved in the onset of autophagy [26]. It is important to be aware that Beclin1 is inhibited through its bindings with the apoptosis inhibitor B-cell lymphoma protein 2 (Bcl2) and caspase-mediated cleavage can inactivate Beclin1-induced autophagy, suggesting the involvement in the crosstalk between apoptosis and autophagy [26-29].

**Figure 1:**
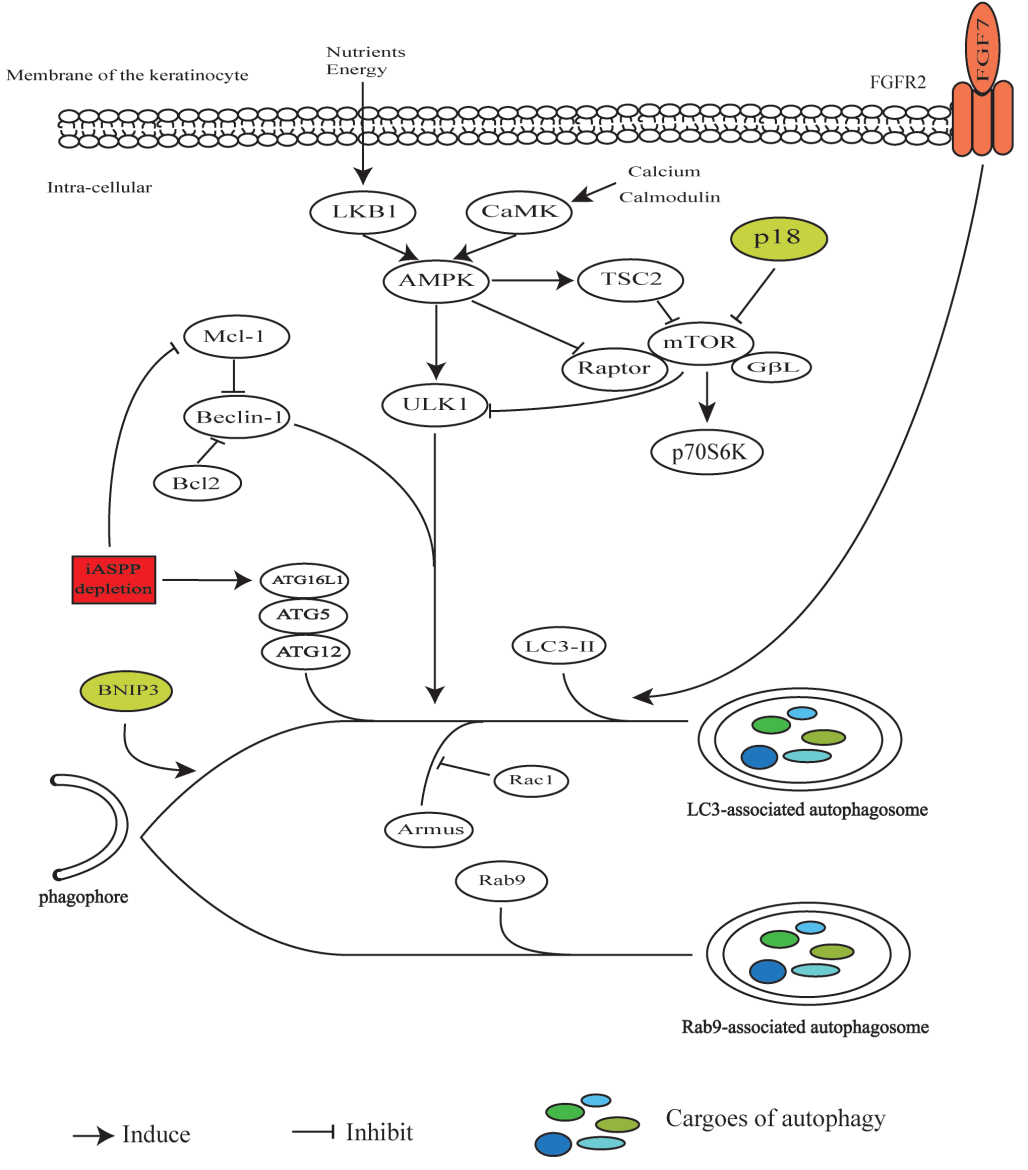
Canonical and non-canonical autophagy pathway in keratinocytes In response to changes of nutrients, energy and intracellular calcium and calmodulin, LKB1 and CaMK activate AMPK following direct modulation of downstream TSC2, raptor and ULK1. The mTOR inhibited by TSC2 or p18 is accompanied with inhibited p70S6K. Beclin1 promoting autophagy process while is inhibited through its bindings with Bcl2 or Mcl-1. ATG5–ATG12–ATG16L1 complex and LC3-II engage in the process from initiation of phagophore elongation to autophagosome formation. The iASPP depletion represses Mcl-1 or promotes ATG5–ATG12–ATG16L1 action. Armus promotes initiation of phagophore which could be blocked by Rac1. Rab9 could be assembled to double-membrane vesicles independently of ATG5/ATG7 and LC3. FGF7-FGFR2 signal stimulates the processes of formation and turnover of autophagosomes. BNIP3 promotes autophagy process but without recognized molecular target yet.

Besides the canonical autophagy pathway, the ATG5/ATG7-independent alternative process of autophagy was also described in keratinocytes [30]. ATG7 blocking in mouse models has no functional consequence for skin development, suggesting that it might be not workable to impair autophagy machinery by simply inhibiting one autophagy-related gene, or there might be an alternative pathway [31]. The non-canonical autophagy pathway with the characteristic of Rab9-positive double-membrane vesicles, which is ATG5/ATG7- or LC3- independent but Beclin1-dependent, was observed in keratinocytes, and a major block or impairment of canonical autophagy pathway has no effect on the expression of Rab9 [30]. The canonical and non-canonical autophagy pathway in keratinocytes with molecule effectors repressing or promoting autophagy process which would be introduced later were described in Figure 1.

## AUTOPHAGY AND KERATINOCYTE BIOLOGY AND PATHOLOGY

Epidermis mainly consists of multilayered continuously renewing keratinocytes, and this structure forms the epidermal barrier which necessarily contributes to the defensive responses against various environmental stimulators, such as heat, cold, trauma, radiation and infection. Increasing findings indicate that autophagy plays an important role on keratinocyte biology and pathology, and involves in keratinocyte-related cutaneous disorders, including psoriasis, zoster herpes and cutaneous squamous cell carcinoma (SCC) [32-35].

### Role of autophagy in keratinocyte differentiation

Differentiation is considered as a special way to death for keratinocytes and essential for the maintenance of tissue homoeostasis. The terminal differentiation in keratinocytes is distinctive and usually named as keratinization. The markers of differentiation such as expression of keratin and involucrin and decrease of β1-integrin appear in different steps of the keratinization [27]. Sequential differentiation or keratinization is accompanied by the degradation of intracellular constituents and activation of lysosomal enzymes [36]. The differentiation-specific expression and localization of LC3 exhibit in keratinocytes [37]. Series of studies based on autophagy inhibition confirmed the role of autophagy in epidermal keratinization process.

Histochemically, the ATG7-deficient mice with low autophagy level showed reduction in diameter and number of keratohyalin and trichohyalin granules, decreased filaggrin, thickened outer root sheath, acanthosis and hyperkeratosis, suggesting that autophagy in keratinocytes participates in differentiation of epidermis [38]. In further, keratinocytes treated with 3-methyl-adenine (3-MA), an inhibitor of autophagy, or of ATG5-depletion showed impairment of differentiation [30]. 3-MA could also block the reversal process of differentiation caused by metabolic stress-induced autophagy [27]. However, the ATG7-deficient mice are resistant to dye penetration into skin and normal transepidermal water loss, indicating that the constitutively active autophagy in epidermis is not essential for skin barrier function [31].

Several proteins were reported recently to involve in regulation of keratinocyte differentiation targeting on distinct autophagy machinery effectors. Investigation on these proteins contributes to integrate autophagy signaling controlling differentiation, even though some of these proteins have not been revealed for recognized molecular targets. Fibroblast growth factor 7/keratinocyte growth factor (FGF7/KGF) induced autophagy in human keratinocytes via phosphatidylinositol 3-kinase (PI3K)-Akt-mTOR pathway, which was proved by the evidence that autophagy depletion counteracted the FGF7-enhanced increase of early keratinocyte differentiation [39]. The mTORC1 is speculated the target of p18 protein, a novel membrane adaptor. The p18-depleted epidermis exhibits severe defects on stratum corneum development and corneocyte formation, and these immature corneocyte-like cells displayed lots of autophagosomes [40]. Normal keratinocytes are of high autophagic activity, and the autophagosomes are rapidly degraded by fusion with lysosomes. Analyses of p18-ablated keratinocytes in the presence of bafilomycin (a V-ATPase inhibitor interrupting autophagosome-lysosome fusion step) revealed that the p18-related complex is required for the formation of autolysosomes by facilitating lysosomal fusion. Furthermore, considering the involvement of p18 in the activation of mTORC1, and the target of p18 is lysosome function, a description on p18-mTORC1 signal function is almost certain [41].

The iASPP protein, a member of ASPP (apoptosis stimulating protein of p53) family, negatively modulates function of p53. The potential link between the iASPP-facilitated autophagy and differentiation in keratinocytes was revealed. Down-regulation of iASPP increased the metabolic form of autophagy against cell death with according changes of a series of autophagic markers including mTORC1, ULK1, Beclin1, p62 and LC3. Furthermore, keratinocytes silenced for iASPP generated thicker epidermis [30]. In the basal layer of epidermis, ATG5–ATG12 partially colocalizes with iASPP which physiologically represses the interaction of ATG5–ATG12 with ATG16L1. Additionally, Bcl2 and adenovirus E1B 19-kDa-interacting protein 3 (BNIP3), an atypical pro-apoptotic BH3-only protein, was described to promote differentiation of keratinocytes by inducing autophagy machinery [42].

### Role of autophagy in melanin metabolism

Epidermal melanin determines the skin color which is associated with ethnic diversity. The melanosomes originated in melanocytes are the main organelles in charge of the melanin biosynthesis. The mature pigmented melanosomes then are transferred into neighboring keratinocytes. Actually, the rates of melanosomes transfer and degradation in keratinocytes determine skin color rather than the melanosome biosynthesis in melanocytes [43, 44]. The autophagy in keratinocytes is the primary mechanism contributing to the skin color development by regulating melanosome degradation. Besides the conventional function of removing the defective melanosomes from melanocytes, autophagy protects cells from melanosome-generated toxicity through eliminating melanosomes in keratinocytes [43, 44]. For instance, it was observed that melanosomes were engulfed into phagosomes in the keratinocytes of the lesion of hypomelanosis [44]. As the distribution patterns of the melanosomes in keratinocytes partly determine skin color, autophagy may play a prominent role in hypomelanosis. Modulating the autophagy machinery might be a better strategy than the melanin synthesis or transfer for treating skin pigmentation disorders or skin cosmetology.

**Figure 2:**
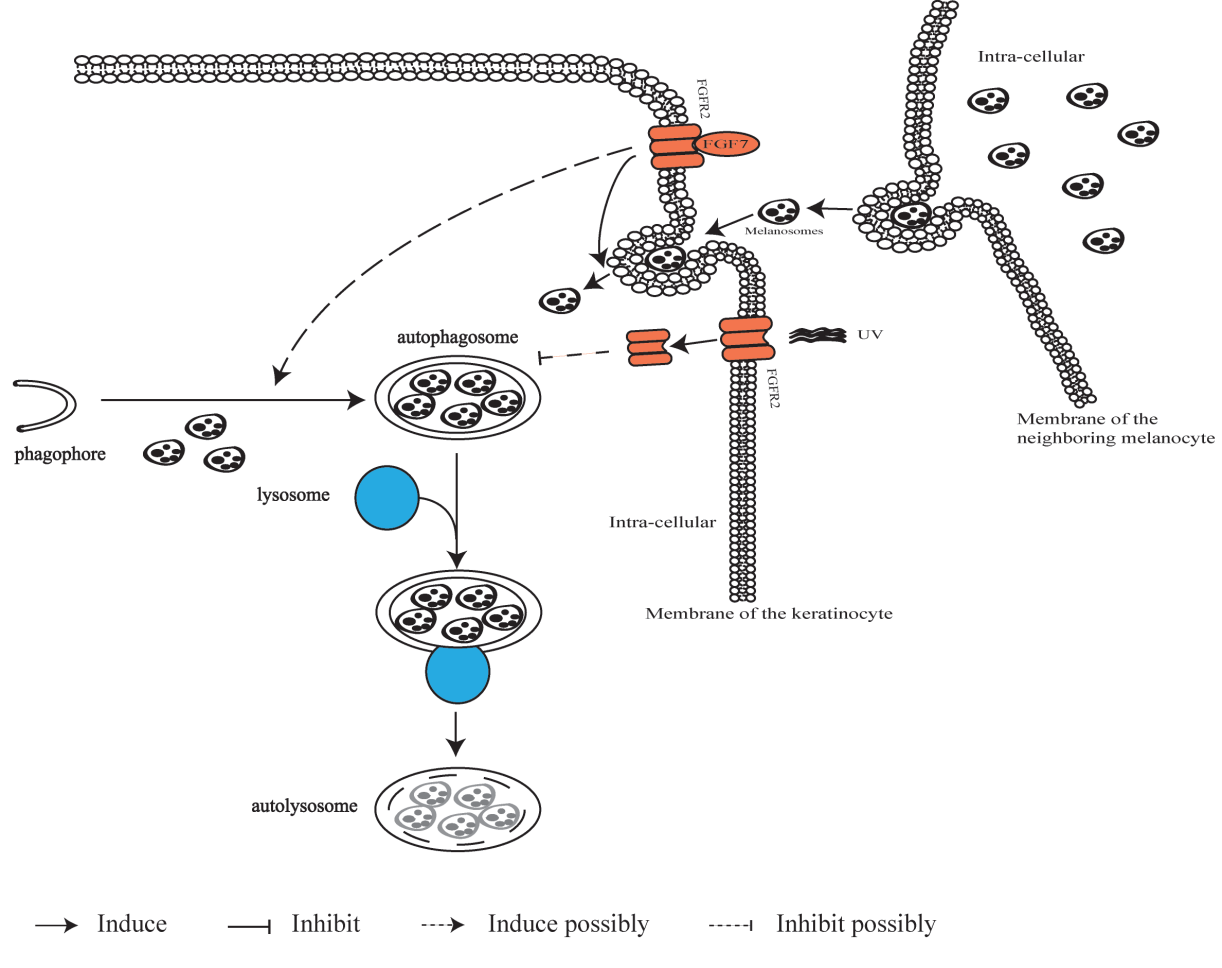
Autophagy machinery in melanin metabolism event Pigmented melanosomes originated in melanocytes are transferred into neighboring keratinocytes, and the entering process is related to activation of FGF7-FGFR2 signal. UV radiation promotes FGFR2 internalization, and subsequent degradation of FGFR2 by autophagosomes/autolysosomes would ease autophagy capacity to degrade melanosomes.

It was reported that FGF7-FGF7 receptor (KGFR/FGFR2) signal involves in skin color regulation, considering that FGFR2 is more expressed in the light keratinocytes (cells derived from light skins) and melanosome uptake is in a FGF7-dependent way [45, 46]. It is interesting that the FGF7-FGFR2 signal is able to promote the autophagy process through stimulating the formation and turnover of autophagosomes [39]. In addition, ultraviolet (UV) B-induced persistence of melanosomes in keratinocytes could be due to the eased autophagy after internalization and degradation of FGFR2 [47]. Figure 2 describes the transferring event of melanosomes originated in melanocytes to neighboring keratinocytes and relevant signaling involving in the activation of autophagy machinery in charge of melanosome degradation in keratinocytes.

### Role of autophagy in immune response

As the accumulation of damaged intracellular organelles like mitochondria and invasive pathogens can cause inflammation, it is not surprising that autophagy functions serves as an anti-inflammatory machinery by eliminating dysfunctional organelles, microbes and viruses, and participates in immune response. For example, overexpression of some inflammatory cytokines, cathelicidin/LL-37 and S100A7 (psoriasin) is related to inhibition of autophagy [48, 49].

**Figure 3:**
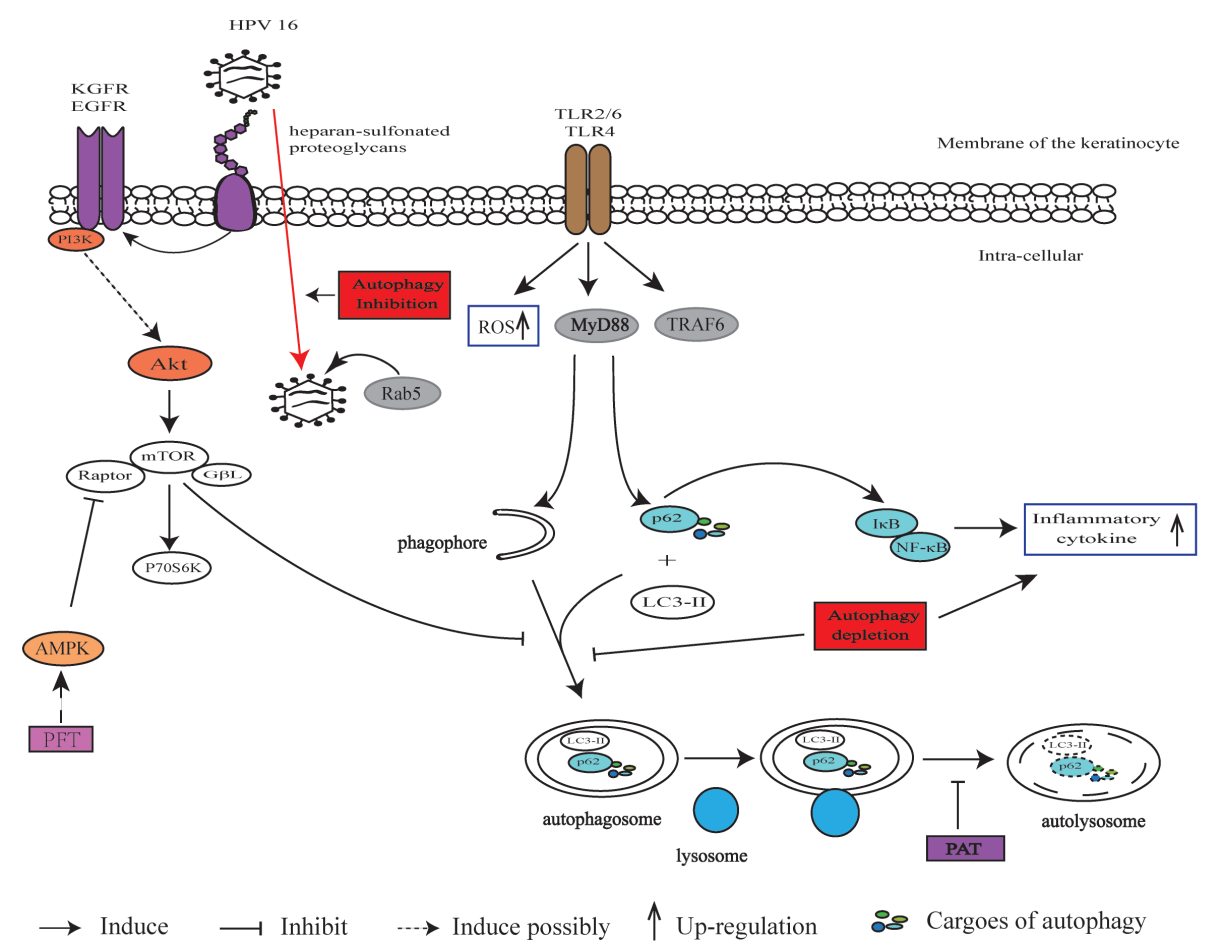
Autophagy machinery in immune response in keratinocytes Activated TLR2/6 and TLR4 led to increased p62 and activated autophagy through generation of ROS, MyD88 and TRAF6. The p62 promotes inflammatory cytokine generation via NF-κB. EGFR or KGFR is in charge of sensing stimulation from HPVs. HPV virus bind to heparan-sulfonated proteoglycans of cell surface and move to EGFR or KGFR following activation of downstream PI3K/Akt/mTOR pathway. During transport HPVs undergo Rab5 association. PFT induces autophagy with a possible target of AMPK. PAT treatment inhibits autophagosome degradation.

Toll-like receptors (TLRs), a group of key recognition molecules in immunity system, involve in the production of antimicrobial immune and pro-inflammatory mediators by activating downstream signals like nuclear factor κ B (NF-κB) and extracellular regulated protein kinase 1/2 (Erk1/2). A number of TLRs are constitutively expressed or inducible in keratinocytes, and the over-expression of some TLRs is related with chronic cutaneous inflammatory diseases such as atopic dermatitis, psoriasis and acne vulgaris [50, 51]. It was reported that TLRs can mediate the cross-link between autophagy and immune signaling in keratinocytes. For example, the activated TLR2/6 led to an increase of p62 expression and autophagy level, and some components in TLR/NF-κB signal such as myeloid differentiation primary response protein 88 (MyD88) and TNFR-associated factor 6 (TRAF6) were necessary for this induction effect [48]. The scaffold protein p62 involves in triggering TLR-induced inflammation as the down-regulation of p62 reduced the activity of NF-κB promoter and expression of cathelicidin. Mediator role of autophagy machinery in transferring TLR signal to the inflammation pathway effector was described in Figure 3. Considering that expression of p62 and TLRs was significantly increased in psoriatic skin, it is highly possible that autophagy participates in this disease and many other cutaneous disorders associated with inflammations.

Furthermore, activation of autophagy machinery in immune response is meaningful to mitochondrial homeostasis. S100A7 (psoriasin) is distributed in the cytoplasm of normal and terminally differentiated keratinocytes, and plays an important role in the cell surface defense for its antimicrobial activity. The over-expression of S100A7 is related to some epidermal inflammatory diseases and skin cancers [52]. The over-expression of S100A7 reversed the increase of Beclin1 and LC3B expression in the presence of LPS in keratinocytes, suggesting that the inhibitory effect of S100A7 on autophagy might be somewhat relevant to the accumulation of damaged mitochondria under lipopolysaccharide (LPS) treatment, as S100A7 is important for mitochondrial biogenesis [49]. Thus moderating keratinocyte autophagy to reduce inflammation may have important implications for the development of novel therapeutic strategies to improve inflammatory disbalance in psoriasis therapy.

### Role of autophagy in antimicrobial defense

As we discussed above, autophagy is regarded as the mechanism to remove damaged organelles and recycle nutrients. It is now well accepted that the keratinocytes serve as the primary component cells of epidermal barrier, and autophagy in keratinocytes involves in host defense against exogenous pathogens and contributes to the innate immune defense by eliminating intracellular viruses and microbes. In contrary, activated autophagy can also be “deceived” by the mutants of some microbes to evade the activation of caspase-1 and inflammasome, and these mutants resist the keratinocyte-mediated clearance [53].

Human papillomaviruses (HPVs) are non-enveloped double-stranded DNA viruses preferably infecting mucosal and cutaneous tissues with keratinocytes as the main targets. HPV infection is responsible for the anogenital cancer and parts of oropharyngeal SCC. HPV16 is the most prevalent pathogenic one among over 100 genotypes of HPVs, of which the infectivity differs among cell kinds, even among different keratinocytes [54]. In the HPV16-infected keratinocytes, autophagy functions as the host defense mechanism to inhibit infection but also plays the part of “accomplice” for cell death. Firstly, considering the importance of Rab5 in the biogenesis and coordination of autophagosomes, HPV16 virions may pass through autophagosomes during entry into the host which is proved by the colocalization of the HPV16 virions with Rab5-containing compartments [55]. Secondly, autophagy inhibited by 3-MA or ATG7-knockdown could significantly enhance the infectivity of HPV16 and delayed the degradation of HPV16 capsid proteins [54, 130]. The degradation is not completely blocked by autophagy inhibition, thus the alternative routes for the trafficking of HPV16 in the host cytoplasm cannot be excluded. Finally, if the persistent autophagy exceeds a threshold, the cell death would occur. In keratinocytes, HPV16 E7 oncoprotein expression primarily mediates an increase of energy requirements due to aberrant cell proliferation or metabolic stress, which leads to the prolonged autophagy process and the cell death under conflicting growth signals [56]. Suppressed autophagy is necessary for the infected cells to survive, which is proved by the experiment of depleting viral early genes [57].

Since the tight involvement of autophagy in internalization and survival of HPVs was proved, the molecular mechanism is put forward. HPVs firstly bind to heparan-sulfonated proteoglycans of cell surface, following move to secondary receptors for internalization [54]. In keratinocytes, epidermal growth factor receptor (EGFR) and keratinocyte GFR (KGFR) are in response for sensing and transferring signals from HPV16 infection to activate the downstream pathways, of which the event is even prior to viral early protein expression [58-60]. As the infection process of HPVs primarily depends on autophagy inhibition, its infectivity might be directly enhanced by inhibiting the autophagy machinery. For example, 3-MA treatment could enhance infection levels, whereas tamoxifen abolished it [58].

Autophagy machinery also participates in bacteria and fungus infection on keratinocytes. Many pathogenic bacteria could produce the virulence factors of pore-forming toxins (PFT) for infection. The membrane perforation is necessary for bacteria infection, and the accompanying efflux of ion would trigger sensors of starvation and energy depletion causing autophagic defense for recovery from sublethal attack. In HaCaT cells (an immortalized keratinocyte cell line), activation of AMPK, subsequent de-phosphorylation/ re-phosphorylation kinetics of S6K and enhanced LC3 lipidation after toxin infection were observed. Formation of LC3-postive puncta and lipid modified form or replenishment of ATP were also inhibited by 3-MA in the alpha-toxin treated cells [61]. Fungus of Aspergillus and Penicillium produce patulin (PAT) for infection. PAT is a mycotoxin and shows carcinogenesis when associated with 12-tetradecanoyl phorbol myristate acetate (TPA) [62]. PAT treatment caused the inhibition of autophagosome degradation accompanied by the accumulation of p62 and consequent reactive oxygen species (ROS) generation in keratinocytes, ultimately disrupting apoptosis via Erk1/2 signaling [63]. Inhibited autophagy by PAT was also associated with reduced activities of lysosomal enzymes cathepsin B/D. The pro-survival signaling initiated by PAT-inhibited autophagy might contribute to the PAT-induced skin carcinogenesis. Autophagy-related signaling in response to HPV, PFT and PAT infection are described in Figure 3.

### Role of autophagy in stress response

As a housekeeping pathway, autophagy is vital for cells to survive various stresses such as nutrient deprivation, oxidative stress, hypoxia and pathogens [64]. Even though autophagy in response to UV is probably due to the deuterogenic oxidative stress, autophagy against UV radiation will be discussed individually.

UV, an extremely common stressor from environment, leads to a series of morphological, ultrastructural and physiological alterations in human epidermis. Keratinocytes respond to the UV-induced damage either by repairing or tolerating it, ultimately undergoing programmed cell death. The electron microscopy result did not show apoptotic cells but the features of autophagy in the UV-treated cells suggesting that autophagy might be one of the cytoprotective mechanisms against the UV-induced apoptosis in keratinocytes [65]. Once some damaged cells escape apoptosis, autophagy may become vital to block canceration. The cells from SCC present higher autophagy level compared with normal keratinocytes, suggesting that the UV stress-induced autophagy might make sense in promoting carcinogenesis and tumor cell survival [66].

UV radiation is classified into UVA, UVB and UVC based on the wavelength, among which UVB is the main cause of skin damage. UVB could induce autophagy of human epidermal keratinocytes at earlier time points [66]. UVA could also induce autophagy in primary murine keratinocytes, but it seems that the UVA-induced autophagy plays an opposite effect compared to UVB. The ATG7-deleted keratinocytes irradiated by UVA showed the defective clearance of p62 and the elevation of nuclear factor-like 2 (Nrf2) target gene expression [67]. As the release of Nrf2 from cytosolic anchor usually cause tissue damage rather than protective responses, the UVA-induced autophagy is highly possible to promote cell death.

Oxidative stress enhanced autophagy is fatal to keratinocytes, which is demonstrated by the decreased death of senescent cells in the presence of 3-MA or anti-ATG5 siRNA treatment [68]. Even though organelles were damaged in the senescent cells, metabolic activity maintains somehow, as mitochondria increased in number. Notably, the apoptosis and autophagy induced by oxidative stress could be inhibited by nacetylcysteine (NAC), a ROS scavenger, indicating that ROS acts as the intermediate for apoptosis and autophagy. Meanwhile, glutathione (GSH) could mimic the protective effect of NAC [69].

Autophagy is also activated in response to stresses induced by hypoxia or ischemia followed by reoxygenation to remove non-functional or damaged cellular structures [70-72]. As we know, re-epithelialization which involves proliferation, differentiation and migration of the keratinocytes is critical for would healing process. Remifentanil, a drug widely used for general anesthesia induction and analgesia, could induce autophagy in keratinocytes and is proved to be beneficial to wound healing against hypoxia-reoxygenation injury. Remifentanil could also recover the 3-MA-blocked autophagy which reveals the possible involvement of autophagy in would healing [73]. Lifting autophagic flux caused by hypoxia in the HaCaT cells also reduced the sensitivity of tumor necrosis factor-related apoptosis-inducing ligand (TRAIL), a toxicant against malignant cells, indicating that autophagy inhibitors could be employed combing with TRAIL in therapies against skin cancers [74].

### Role of autophagy in keratinocyte senescence

Senescence is described as a kind of programmed cell death. The senescent cell with its own time schedule is cell-cycle arrested and displays morphological, metabolic and genetic changes differing from the dead or young one. Cell death now is classified into three types: apoptosis (type I), autophagy programmed death (type II) and necrosis (type III) [75, 76]. The senescent keratinocytes usually present extremely high autophagic activity characterized by an accumulation of a large number of autophagy vacuoles and a particular intracellular organization [77]. The autophagic programmed cell death is the primary mechanism of cell death for the senescent keratinocytes rather than apoptosis. Death of senescent cells was delayed when the initial phase of autophagy was blocked by 3-MA, without any change on apoptotic markers, meanwhile apoptotic inhibitors could not decrease the cell death in senescent cells [77]. However, apoptosis is likely to co-exist in low proportions with autophagy, of which the significance need to be clarified. Apoptosis may compromise autophagic cell death against oxidative stress, or increased autophagy might initiate apoptosis. Advancing age, telomere erosion and oxidative damage can lead to senescence [78]. Oxidative stress might be the key incentive factor. Intracellular accumulation of H_2_O_2_ could enhance autophagy to eliminate the oxidative-damaged mitochondria and nuclei, and initiate the senescence in keratinocytes and the program for final death [68].

In this sense, clarifying the role of autophagy in senescence would be of pathological and physiological significance. Then autophagy and senescence share the same marker of senescence-associated β-galactosidase (SA-β-Gal) which is increased during aging [79, 80]. Together with the fact that oxidative stress could accelerate aging, we consider that there should be inevitable links among autophagy, senescence and aging.

Recently, it was reported that the Ras-Raf-Erk-mediated autophagy and senescence is essential to prevent the skin tumorigenesis. Xie *et al*. [81] discovered that the activation of Kras initiated Erk signaling to induce a high level of ROS production, which down-regulated the activity of mTORC1 to enhance autophagy and senescence in the keratinocytes with normal protein expression of Sag. However, Sag deletion led to the accumulation of Erbin and Nrf2, two substrates of Sag, and subsequently caused the blockage of Ras-Raf signaling and clearance of ROS respectively. These events resulted in the relief of mTOR inactivation and blockage of autophagy and senescence. Accordingly, accelerated papillomagenesis has been observed in the skin of the Sag^−/−^ mice. Since escaping senescent-cell death might be a requisite step for tumor formation, application of possible autophagy inducers responsible for autophagic programmed cell death might be a therapy strategy through eliminating senescent cells.

### Autophagy determines keratinocyte fate, to survive or to die?

In most instances, autophagy functions as a survival processor, though it may turn into the lethal machinery when inappropriate level of autophagy occurs. Cells digest themselves to resist adverse factors like metabolic stress induced by nutrient deprivation or energy deficiency. When autophagy is incapable to eliminate intracellular damage it may shift to the programmed cell death. For instance, autophagy will lead to a destructive outcome when parallel damage in lysosomal and mitochondrial membranes occurs [82]. Autophagic programmed cell death is characterized by accumulation of numerous autophagosomes and incontinent degradation of cytosolic components, whereas the nucleuses remain intact until late stage [83]. Even though beyond thirty ATG members have been reported to involve in the autophagy process, they act differently during “survival” autophagy and the “death” one. For example, levels of ATG5 and Beclin1 do not change during the starvation-induced autophagy but increase during the autophagic programmed cell death [84]. Conflicting with this finding, there comes an interesting phenomenon in the keratinocytes that Beclin1 indeed increases in comparison to the normal cells, even though the keratinocytes are of high basic autophagic activity which is enough for senescent cells to end up by death [68].

Both autophagy and apoptosis are in response for cell death as we mentioned before, and the two processes are intimately connected. In keratinocytes, activated autophagy plays an indispensable role of anti-apoptosis to promote cell survival against various stresses. For example, autophagy deficiency facilitates the activation of p38 pathway and subsequent apoptosis suggesting the anti-apoptotic action of autophagy [84]. A possible switch controlling autophagy and apoptosis in keratinocytes is recently described. In the canonical autophagy pathway, the increase of autophagy is accompanied by the dissociation of Beclin1 from myeloid cell leukemia-1 (Mcl-1) [85]. Mcl-1 can complex with Noxa, a pro-apoptotic effector belonging to Bcl-2 family, of which the activation trigger apoptosis after UVB irradiation in a p53-dependent or independent manner [86]. In the iASPP-depleted keratinocytes, the increase of Beclin1 and decrease of Mcl-1 and Noxa have been observed simultaneously [30]. Thus iASPP expression is proposed a switch controlling autophagy and apoptosis in this cell setting.

## THERAPEUTIC APPROACHES FOR SKIN DISEASES

### Resveratrol

Trace back to 1990s, resveratrol was reported to have protective effect on skin against the UV-induced photodamage and photoaging [87]. Then further studies implicated the involvement of resveratrol in the regulation of autophagy partially due to its effects on redox balance [88]. Autophagy induced by resveratrol pre-treatment alone can be revealed by the reversible and irreversible suppression on the Akt-mTOR signaling and the increase of Beclin1 expression as well as the cell growth arrest which conforms to the canonical pro-survival autophagy in HaCaT cells [89]. However when associated with UVB of different dosages, the autophagy converted to a fatal one. Enhanced apoptosis and autophagy coexist in the keratinocytes pre-treated with resveratrol before UVB irradiation reducing the odds of non-tumorigenic cells to escape death. Because of the ability to interfere with multiple cellular pathways, resveratrol is promising for the prevention and therapy of hyperproliferative skin diseases like malignant cancers. For example, the premature senescence induced by resveratrol was associated with autophagic blockage via Rictor, a component of mTORC2 [90] in A431 epidermoid carcinoma cell line (malignant keratinocytes derived from SCC).

### Calcipotriol

Calcipotriol, an analog of vitamin D, could induce autophagy in both normal and immortalized keratinocytes [91]. The vitamin D analogs have been used for the treatment of many skin diseases including psoriasis, recalcitrant warts, lamellar ichthyosis and epidermolytic hyperkeratosis [92-94]. Now recognizing the regulatory mechanism of calcipotriol on autophagy would be helpful to understand the pleiotropic effects of vitamin D analogs and vastly expand the application on the therapy for some skin disease, for example intracellular infection and hyperproliferation.

### α-Santalol

α-Santalol, one of the primary components of sandalwood oil which commonly used as fragrances and incense, has been reported to have potential tumor prevention effect against UV exposure. α-Santalol treatment could induce autophagy along with an increase of impaired plasma membrane integrity in the HaCaT cells, but not apoptosis [95]. Since the effect is more prominent in the proliferating cells than in the quiescent cells, cytotoxic treatment might be considered as an alternative way for the skin cancer therapy, which exerts less damage on normal cells via selective induction of autophagy.

### Apigenin

Apigenin, a kind bioflavonoid, presents in variety of food sources and performs the antitumorigenic and chemopreventive actions [96]. The chemoprotective action of apigenin can be interpreted by the induction of autophagy via CaMK kinase-β (CaMKKβ)-AMPK-TSC2-mTOR and Akt-TSC2-mTOR signaling, but mainly through the activation of the former one in HaCaT cells [97]. Additionally, apigenin pretreatment could dramatically enhance the UVB-induced AMPK activation while UVB treatment alone just exerted moderate effect.

### Chromium and hexavalent chromium (Cr (VI))

Chromium or hexavalent chromium (Cr (VI)) is ubiquitous in the environment and contributes to some skin diseases like chromium hypersensitivity and allergic contact dermatitis of which the occurrence is related to the ROS-mediated activated autophagy [98, 99]. In HaCaT cells, Cr (VI) treatment increased the LC3-II production, while combining with the pretreatment of NAC, a ROS scavenger, prohibited this effect. Moreover, the study on albino guinea pig model showed that NAC was curative for the chromium hypersensitivity [100]. The regulation details have not been described, but it can be speculated to involve in the Akt pathway.

### 5-fluorouracil (5-FU)

The antimetabolite 5-FU is commonly used in the therapy of colorectal cancer [101]. 5-FU inhibits thymidylate synthase enzyme to block DNA synthesis and also interferes with RNA processing. Similar to many other drugs, resistance is one of the major problems that retarded curative effects, of which the mechanisms involve alterations in related enzymes, p53, ataxia telangiectasia mutated kinase (ATM) or ataxia telangiectasia and Rad3-related kinase (ATR). It was reported that activated p38 or resurgent autophagy mediates the resistance to 5-FU in the HaCaT cells [102]. p38 is activated by 5-FU through both ATM and ATR, and is dependent on its canonical adjacent up-stream signals, such as mitogen-activated protein kinase kinase (MAP2K), MAP kinase kinase 3 (MKK3) and MKK6. In the normal conditions, the genotoxic response triggered by 5-FU treatment promotes apoptosis and blocks autophagy. Nevertheless, when p38 is blocked, the increase of autophagy with the decrease of apoptosis rendered the resistant phenotype. Therefore, 5-FU is a crucial regulator controlling the balance between apoptosis and autophagy to determine cell fate. Levofolene (LF), often used in combination with 5-FU, showed an antagonistic effect on autophagy to modulate apoptosis elicited by 5-FU treatment [103]. Therefore, the inhibition of 5-FU induced autophagy contributed to the enhancement of toxicity on keratinocytes treated with 5-FU in combined with LF treatment, which can produce a severe dermal side effect, for example hand-foot syndrome.

### Efavirenz (EFV)

EFV is commonly used for the antiretroviral therapy, but usually with the adverse drug reaction of cutaneous and mucosal lesions [104]. The keratinocytes exposed to EFV exhibited a decrease of viability and premature terminal differentiation, leading to the impaired epithelial regeneration and subsequent atrophy [105]. Assessment of the related intracellular signaling showed the depressed mTOR signaling and activated Erk1/2 signaling together with rapid p53 degradation, indicating that the inhibitory effect of EFV on epithelial regeneration is related to the autophagy activation.

### Oligosaccharides

Some nature oligosaccharides such as trehalose, sucrose and raffinose were reported to be cargos of autophagosomes. Noticeably, trehalose exhibited a therapeutic effect through an mTOR-independent induction of autophagy in preventing neural tube defects, retarding the progression of amyotrophic lateral sclerosis, alleviating the dopaminergic and tau pathology, resisting the cellular prion infection and promoting the clearance of mutant huntingtin and alpha-synuclein [106-110]. Our recent work revealed that trehalose, sucrose, and raffinose can enhance autophagy in human keratinocytes via the mTOR-independent way, and trehalose treatment decreases cell death and prohibits the abnormal cell proliferation caused by UV stimulation (data have not been published).

## CONCLUSIONS

As a highly conserved intracellular mechanism for homeostasis maintenance, autophagy is proved to participate in series of physiological activities in keratinocytes, including apoptosis, differentiation, inflammation and melanin metabolism. Autophagy is even closely related to senescence, aging, skin color formation, and also displays the ability of anti-stress and anti-infection. Most notably, it distinctly functions as the switch for controlling the cell death, as some times autophagy presents a cytoprotective talent, but it shifts to a fatal mechanism in some conditions. Thus, application of autophagy regulators in keratinocytes implied a promising strategy for the therapy of abundant skin diseases.

However, the cells with defects in autophagy also showed persistent DNA damage and increased chromosome instability, while excessively activated autophagy might also leads to adverse results. Therefore, much works are needed on optimizing designed strategies to inhibit or enhance autophagy for clinical efficacy, since autophagy routinely plays the role of a double-edged sword in various conditions, which can be illustrated by the studies on the performances of autophagy in cancer. On one hand, autophagy acts as a tumor suppressor to clear damaged organelles and accumulated growth factors and maintain chromosomal stability. In this case, Beclin1 and ATG5 have been identified as the “guardian” for the cellular genome [111]. On the other hand, the cytoprotective effect of autophagy on cancer cells through damage elimination and energy supply would be helpful to resist anticancer therapy [112]. Now, autophagy inhibitors and activators were both applied combining with anticancer agents to reinforce the efficiency [113]. Indeed, autophagy not only eliminates the damage caused by anticancer treatment, but also provides the energy for cell division. For example, autophagy is activated in the cancer cells which are in circumstance of hypoxia, a common condition in solid tumor [114]. Under this non-physiological event, autophagy is useful to sustain the cancer cells’ proliferation through degrading proteins, DNA and organelles and maintaining their energy provision. Thus, the issue of how to manipulate autophagy to fight against tumorigenesis remains extremely intriguing.

Series of evidences summarized in this review demonstrated the extremely complicated role of autophagy in cutaneous physiology and pathology. However, there remains questions impeding further clarification of the role of autophagy in keratinocytes: (i) The basal autophagy level is distinct in the different keratinocyte settings like primary keratinocytes and HaCaT cells, as well as epidermal cancer cell line (e.g. A431), possibly due to different culture conditions (culture medium with or without serum) or origins of human races. (ii) Studies in *vivo* were extremely limited, particularly on the performance of autophagy in common skin disorders associated with keratinocytes. (iii) Unique autophagy machinery probably exists in human keratinocytes.

About forty ATG genes have been identified in the mammalian cells and/or yeast without complete confirmation of their functions. The ATG proteins in keratinocytes remain to be clarified for their expression regulation, protein-protein interaction, roles in autophagic machinery and extended functions. Recently, Kemp *et al.* reported that ULK1 is deregulated due to the UV-induced DNA damage in keratinocytes, which caused a stimulator of interferon genes (STING)-dependent interferon regulatory factor 3 (IRF3) activation, suggesting the function of ULK1 in immune response [115]. Besides the autophagy regulation, ATG proteins perform other biological functions such as inflammation, immunity and efferocytosis. Therefore, we consider that clarification of distinct performances of the ATG proteins in keratinocytes is essential to demonstrate the biological roles of autophagy in skin disorders.

At last and notably, besides series of signaling effectors we mentioned above, microRNAs (miRNAs), a group of RNA nucleotides with the function of modulating mRNA translation/degradation, emerge as promising targets for cancer therapy, and numerous evidences have indicated the role of miRNAs in cell biology [118, 119]. Recently numbers of miRNAs were found exposing their tight links to autophagy pathway. For instance, autophagy contributes to miRNA activity and homeostasis by regulating the degradation of cytosolic miRNA-containing complexes or modulating miRNA-mediated gene silencing [120, 121]. Accordingly, miRNAs were reported to involve in autophagy regulation by directly targeting proteins associated with autophagy like Beclin1, Atg4C, ATG5, FAK family-interacting protein of 200 kDa (FIP200), ATG7 and even mTOR [122-124]. However, there is very a few reports yet on interaction performance of miRNAs and autophagy in keratinocytes, most studies devoted to understanding miRNA performances in keratinocyte proliferation, migration, differentiation and senescence and without description of autophagy involvement [125-127]. It's exciting that if combing with available results we have described in this review above, these limited results would be inspiring. For instance, Rivetti *et al*. reported the contribution of miR-138, −181a, −181b and −130b to induction of senescence in keratinocytes by modulating levels of p63 (belong to p53 family) and silent mating-type information regulation 2 homologue 1 (Sirt1) [126]. Considering the interplay between p53 or Sirt1 and autophagy, and the relationship between autophagy and senescence in keratinocytes have already been revealed, we could speculate that autophagy could act the intermediation role between miRNAs and senescence [27, 30, 105]. Conclusively, considering the widespread significance of miRNAs in cell biology regulation and increasing evidences revealing the tight involvement in autophagy, clarifying relationship between miRNAs and autophagy in keratinocytes is needed more investigation in depth.

Drugs targeting autophagy modulation have been followed with the interests in treatment for cancer, infection and neurodegenerative diseases. For instance, autophagy blocker chloroquine and its analogue of hydroxychloroquine have been exploited in series clinical trials to potentiate anticancer therapy [128, 129]. Importantly, chloroquine and hydroxychloroquine are widely used in the therapy for many kinds of skin disorders such as photosensitivity dermatosis, lupus erythematosus and lichen planus. However, it remains to confirm whether autophagy affected by treatment of them is related to the therapeutic effect against these diseases. In a word, the agents targeting autophagy modulation are extremely potential for widely application in dermatology in the individual or combination manner.
